# Re-evaluation of microscopy confirmed *Plasmodium falciparum* and *Plasmodium vivax* malaria by nested PCR detection in southern Ethiopia

**DOI:** 10.1186/1475-2875-13-48

**Published:** 2014-02-06

**Authors:** Seleshi Kebede Mekonnen, Abraham Aseffa, Girmay Medhin, Nega Berhe, Thirumalaisamy P Velavan

**Affiliations:** 1 Aklilu Lemma Institute of Pathobiology, Addis Abba University, Addis Abba, Ethiopia; 2 Armauer Hansen Research Institute, Addis Abba, Ethiopia; 3 Institute of Tropical Medicine, University of Tübingen, Wilhelmstraße 27, 72074 Tübingen, Germany

**Keywords:** Malaria, *Plasmodium*, Nested PCR, Microscopy, Ethiopia

## Abstract

**Background:**

With 75% of the Ethiopian population at risk of malaria, accurate diagnosis is crucial for malaria treatment in endemic areas where *Plasmodium falciparum* and *Plasmodium vivax* co-exist. The present study evaluated the performance of regular microscopy in accurate identification of *Plasmodium spp*. in febrile patients visiting health facilities in southern Ethiopia.

**Methods:**

A cross-sectional study design was employed to recruit study subjects who were microscopically positive for malaria parasites and attending health facilities in southern Ethiopia between August and December 2011. Of the 1,416 febrile patients attending primary health facilities, 314 febrile patients, whose slides were positive for *P. falciparum*, *P. vivax* or mixed infections using microscopy, were re-evaluated for their infection status by PCR. Finger-prick blood samples were used for parasite genomic DNA extraction. Phylogenetic analyses were performed to reconstruct the distribution of different *Plasmodium spp.* across the three geographical areas.

**Results:**

Of the 314 patients with a positive thick blood smear, seven patients (2%) were negative for any of the *Plasmodium spp*. by nested PCR. Among 180 microscopically diagnosed *P. falciparum* cases, 111 (61.7%) were confirmed by PCR, 44 (24.4%) were confirmed as *P. vivax*, 18 (10%) had mixed infections with *P. falciparum* and *P. vivax* and two (1.1%) were mixed infections with *P. falciparum* and *P. malariae* and five (2.8%) were negative for any of the *Plasmodium spp*. Of 131 microscopically diagnosed *P. vivax* cases, 110 (84%) were confirmed as *P. vivax,* 14 (10.7%) were confirmed as *P. falciparum*, two (1.5%) were *P. malariae*, three (2.3%) with mixed infections with *P. falciparum* and *P. vivax* and two (1.5%) were negative for any of the *Plasmodium spp. Plasmodium falciparum* and *P. vivax* mixed infections were observed. *Plasmodium malariae* was detected as mono and mixed infections in four individuals.

**Conclusion:**

False positivity, under-reporting of mixed infections and a significant number of species mismatch needs attention and should be improved for appropriate diagnosis. The detection of substantial number of false positive results by molecular methodologies may provide the accurate incidence of circulating *Plasmodium* species in the geographical region and has important repercussions in understanding malaria epidemiology and subsequent control.

## Background

Malaria is one of the major causes of mortality and morbidity in tropical and subtropical countries with an estimated 655,000 malaria deaths in 2010, and 91% of these cases were in sub-Saharan Africa [1]. Malaria is caused by parasites of the genus *Plasmodium,* with four different species specifically infecting humans (*Plasmodium falciparum*, *Plasmodium vivax*, *Plasmodium ovale* and *Plasmodium malariae)* and parasites of non-human primates, such as *Plasmodium knowlesi*, infecting humans occasionally. *Plasmodium falciparum* is the most virulent species contributing to a larger extent to malarial deaths in Africa, including Ethiopia. *Plasmodium vivax* and *P. ovale* form resting stages in the liver as hypnozoites and can cause a clinical relapse [2].

Ethiopia’s diverse topography and climatic conditions make malaria a seasonal infection covering 75% of the prefecture of the country [3] and malaria epidemic occur mostly in high and low land arid regions of the country [4]. The majority of Ethiopian regional states (Oromia, Amhara, Tigray, South Nation, and Nationalities, and People’s Region (SNNPR)) have experienced the 1998 malarial epidemic [5]. *Plasmodium falciparum* accounts for 60-70% and *P. vivax* accounts for 30-40% of malaria cases in Ethiopia, although these proportions might fluctuate on the spatial and temporal scale [6]. *Anopheles arabiensis* is the main vector widely distributed in Ethiopia, while *Anopheles pharoensis*, *Anopheles funestus* and *Anopheles nili* contribute as secondary vectors [7]*.*

Hospitals and health centres in Ethiopia diagnose malaria by microscopy, whereas the health posts employ rapid diagnostic tests (RDT) for diagnosis of *P. falciparum* and *P. vivax*[3]. The use of RDTs in malarial diagnosis does not require skilled personnel compared to microscopy and reduces inappropriate use of anti-malarial drugs [4]. However, RDTs do not allow quantification and differentiation of different *Plasmodium* species other than *P. falciparum* and *P. vivax*. Microscopy remains the golden standard of choice to determine the disease burden. Additionally microscopy can identify febrile patients negative for any *Plasmodium* infection, thereby directing patients towards an alternative treatment algorithm [5,8].

Microscopy is a valuable technique and has been shown to detect about 75% of malaria infections in high transmission areas and miss up to 88% of infections in low transmission areas [9]. The detection of low numbers of parasites, and the labour-intensive procedure remain a hurdle in differentiating *Plasmodium* species by microscopy [10]. In endemic regions, *P. malariae* and *P. ovale* infections are frequently overlooked. Dedicated laboratory personnel with good experience remain the key for accurate diagnosis of *P. malariae* and *P. ovale* in areas where *P. falciparum* and *P. vivax* predominate. Prompt and accurate diagnosis followed by an effective treatment largely depends on the skills of the laboratory personnel, who can spend appropriate time in examining blood film. On the other hand, microscopy techniques fail to detect mixed infections, when one of the *Plasmodium* species is present at low levels.

In recent years, molecular detection of *Plasmodium* with higher sensitivity is gaining importance for accurate detection. Methods such as nested PCR are capable of detecting few parasites/μl in blood [10-15]. The nested PCR approach identifies the species-specific *Plasmodium* DNA by amplifying the 18s ribosomal RNA region of the parasite [11]. The ribosomal RNA genes in *Plasmodium* are four to eight copies per haploid genome and are scattered on different chromosomes, with two distinct subgroups whose expression is regulated both by type A and type B genes that are expressed in the asexual and sexual stages in the vertebrate host, respectively [16,17]. The nucleotide sequence of the SSUrRNA is largely conserved between *Plasmodium* and different species reveal genetic heterogeneity in their respective ribosomal regions. This variation, together with the abundance of ribosomes in the parasite, has led to the development of effective diagnostic probes based on SSUrRNA sequences [18].

In Ethiopia, previous reports on malaria surveillance were based on microscopic results. The majority of these studies were conducted either with little or no information or with few technical resources [19-21]. In the current study, the accuracy of results obtained by routine microscopy performed by experienced laboratory personnel was compared to molecular detection methodologies. These microscopists were experienced and were supervised by fellow senior laboratory technologists. In addition, they received additional training on a regular basis to improve their detection skills on differentiating *Plasmodium spp*. Additionally, 18SrRNA genes were subsequently sequenced and phylogenetic relationship was investigated to determine the genetic diversity of the *Plasmodium spp*.

## Methods

### Study area and design

A health institution-based study targeting febrile patients attending primary health facilities was conducted in southern Ethiopia between August and December, 2011. Malaria transmission in Ethiopia is seasonal with higher transmission peaks in the months between September and December, and between April and May following rainy seasons. The three study sites included in this study were Omo Nada, Bala Wajo and Arba Minch.

### Study participants

Study participants were recruited from the routine health delivery system. Self-presenting febrile patients attending health centres in Omo Nada (n = 713), Bala Wajo (n = 312) and Arba Minch (n = 391) and whose age was at least six months between August and December 2011 were eligible for the current study. Any patient reported as being infected either with *P. falciparum* or *P. vivax* or with a mixed infection using the existing health care delivery system within the target health centres was approached for consent immediately after receiving their laboratory result. For those who consented to be part of the study, approximately 5–10 μl finger-prick blood sample was collected. No additional clinical assessment was carried out at the time of recruitment.

### Sampling

In total, 314 finger-prick blood samples were collected for molecular diagnosis from microscopy-positive outpatients in Omo Nada (n = 132), Bala Wajo (n = 99) and Arba Minch (n = 83) from all patients attending the health facilities during the study period. Blood samples were collected in Whatman 3 mm filter papers. These patients were positive in the routine health delivery system using microscopy either for *P. falciparum* or *P. vivax*. On collection, the blood spots were air-dried and stored in a separate, clean, sealed, plastic pouch at room temperature for further analysis.

### Ethics

Ethical clearances were obtained from the institutional review boards of Aklilu Lemma Institute of Pathobiology, Jimma University and Armauer Hansen Research Institute. Informed written consent and assent (in the case of children between 12 and 18 years of age) were obtained from each patient or a parent/legal guardian. The study participation was voluntary and had no influence on the treatment provision at the respective health facilities. All patient information were kept confidential.

### Blood film management and patient care

Giemsa-stained thick and thin blood films are routinely used in Ethiopian hospitals and health centres for the detection of *Plasmodium* without any quantitative estimation of parasitaemia. In the current study, thin and thick blood films were prepared from finger-prick blood and air-dried, fixed in methanol and subsequently stained for 20–30 minutes in Giemsa. At least 100 thick field films with an oil immersion lens at 100X magnification were examined before a slide was considered negative. All individuals positive for *P. falciparum* were treated with artemether-lumefantrine (AL), *P. vivax-*positive patients were treated with chloroquine and mixed infections were treated with AL.

### DNA extraction and molecular detection

Genomic DNA was isolated using QIAgen DNA Mini Kit blood and tissue (QIAGEN, Germany) according to the manufacturer’s instructions. The extracted DNA was stored at -20°C until use. Nested PCR assay were carried out as previously described elsewhere [20]. DNA samples were amplified by species-specific primer pairs designed to amplify small subunit ribosomal ribonucleic acid (ssrRNA) genes of *P. falciparum*, *P. vivax*, *P. malariae* and *P. ovale* (Table 1). In brief, both the primary and nested amplifications were carried out in a 20 μl reaction volume containing 1X buffer, 2.5 mM MgCl2, 200 μM dNTPs, 200 nM primers, and 1U Taq DNA-polymerase with approximately 10 ng of DNA template on a PTC-200 Thermal cycler (MJ Research, USA). The *Plasmodium* genus-specific amplification was followed by *P. falciparum*, *P. vivax*, *P. malariae* and *P. ovale* species-specific PCR amplification. Thermal cycling parameters for first round of amplification were: initial denaturation at 94°C for 5 min, followed by 40 cycles of, respectively, 25 sec at 94°C for denaturation, 25 sec for 58°C for annealing temperature, and 60 sec at 72°C extension and the thermal parameters for the second PCR were followed by 30 cycles of, respectively, 25 sec at 94°C for denaturation, 25 sec for 56°C for annealing temperature, and 60 sec at 72°C extension. This was followed by a final extension of 10 min at 72°C. In each run negative and positive controls were included. Genomic DNA from healthy as well as from individuals who had not travelled to malaria-endemic areas were included as negative controls in all PCR diagnostic assays. Amplicons were separated on a 1.2% agarose gel electrophoresis run along with a 100 bp DNA ladder (Invitrogen, Karlsruhe, Germany). The presence or absence of different *Plasmodium* species was confirmed with representative amplicon size that were species-specific. Samples that failed to amplify were subjected to repeated amplification procedures with different PCR additives.

**Table 1:** Species-specific primers used for nested PCR and sequencing

**Malaria species**	**Primer**	**Primer sequence 5′-3′**	**Size (bp)**
*Plasmodium*	rPLU5	CCTGTTGTTGCCTTAAACTTC	1,100
	rPLU6	TTAAAATTGTTGCAGTTAAAAC	
*P. falciparum*	rFAL1		250
	rFAL2	ACACAATGAACTCAATCATGACTACCCGTC	
*P. vivax*	rVIV1	CGCTTCTAGCTTAATCCACATAACTGATAC	120
	rVIV2	ACTTCCAAGCCGAAGCAAAGAAAGTCCTTA	
*P. malariae*	rMAL1	ATAACATAGTTGTACGTTAAGAATAACCGC	144
	rMAL2	AAAATTCCCATGCATAAAAAATTATACAAA	
*P. ovale*	rOVA1	ATCTCTTTTGCTATTTTTTAGTATTGGAGA	800
	rOVA2	GAAAAGGACACATTAATTGTATCCTAGTG	

### Sequencing and phylogenetic analysis

Thirty-one (n = 31) *Plasmodium*-positive samples were further selected to determine the genetic diversity of the existing subspecies from three collected sites. The selected *Plasmodium* samples included 11 *P. falciparum* (three from Omo Nada; two from Bala Wajo; six from Arba Minch), 16 *P. vivax* (nine from Omo Nada; four from Bala Wajo; three from Arba Minch) and four *P. malariae* from Omo Nada. PCR products were cleaned up using Exo-SAP-IT (USB, Affymetrix, USA) and 1 μl of the purified product were directly used as templates for sequencing, using the BigDye terminator v. 2.0 cycle sequencing kit (Applied Biosystems, USA) on a ABI 3130XL DNA sequencer, according to the manufacturer’s instructions.

Five 18srRNA-specific DNA sequences for each *Plasmodium* (*P. falciparum* JQ627152.1, *P. vivax* HF945443.1, HF945441.1 and JQ627152.1 and *P. malariae* KC906731.1) obtained from NCBI database were aligned with 31 samples (11 *P. falciparum* samples, 16 *P. vivax* samples and four *P. malariae* samples) sequenced for 18srRNA using Codon code Aligner 4.0 software. The phylogenetic tree was reconstructed by Maximum Likelihood method based on Kimura 2-parameter model using the MEGA ver5.2 software [22] with 1000 bootstrap iterations.

## Results

### Study subject characterization

Blood films were examined from a total of 314 study participants aged at least six months for the presence of malaria parasites, using microscopy as well as by species-specific nested PCR method. Among the study participants, 21% were children less than four years of age, 60.2% were males and 38.9% were females. Of these study participants, 307 were reconfirmed positive for the presence of *Plasmodium* DNA by PCR. Of 314 study subjects, 21% were children less than four years of age, 189(60.2%) were male and 125(38.9%) were female. Among 307 PCR-confirmed cases, 125(40.7%) were positive for *P. falciparum*, 154(50.2%) were positive for *P.vivax*, two (0.7%) were positive for *P. malariae*, 24(7.8%) were positive for *P. falciparum* and *P. vivax* double infections, two (0.7%) were positive for *P. falciparum* and *P. malariae* double infection. Seven study participants remained negative for all species investigated (Table 2).

**Table 2:** Malaria parasite species stratified by sex and age among 314 study participants in southern Ethiopia

		***Pf *** **(%)**	***Pv *** **(%)**	***Pm *** **(%)**	***Pf + Pv *** **(%)**	***Pf + Pm *** **(%)**	**Negative (%)**	**Total (%)**
**Sex**	**Male**	75 (39.7)	91 (48.1)	2 (1.1)	16 (8.5)	1 (0.5)	4 (2.1)	189 (60.2)
	**Female**	50 (40)	63 (50.4)	0 (0)	8 (6.4)	1 (0.8)	3 (2.4)	125 (38.9)
**Age group (years)**	**≤ 4**	29 (43.9)	32 (48.5)	1 (1.5)	4 (6.1)	0 (0)	0 (0.0)	66 (21.0)
	**5-14**	55 (35.7)	81 (52.6)	1 (0.6)	12 (7.8)	1 (0.6)	4 (2.6)	154 (49.0)
	**15-28**	22 (37.3)	28 (47.5)	0 (0)	5 (8.5)	1 (1.7)	3 (5.1)	59 (17.8)
	**29-45**	13 (46.4)	12 (42.9)	0 (0)	3 (10.7)	0 (0)	0 (0.0)	28 (8.9)
	**>46**	6 (85.7)	1 (14.3)	0 (0)	0 (0)	0 (0)	0 (0.0)	7 (2.2)

*Pf = P. falciparum, Pv = P. vivax , Pm = P. malariae, Po = P. ovale, Pf + Pv = P. falciparum* and *P. vivax, Pf + Pm = P. falciparum* and *P. malariae*.

All single and mixed infections of *P. malariae* were not detected by the golden standard microscopic examination. Most of the *Plasmodium*-infected individuals belong to the age group between five and 14 years (n = 154, 49%). Of these 154 individuals aged from 5 to 14 years, 81 (52.6%) were infected by *P. vivax* and 55 (35.7%) infected by *P. falciparum*. A total of 66 (21%) were children less than four years. The ages of all *P. malariae*-positive cases that were detected by PCR were below 28 years (Table 2).

### Comparison of molecular diagnosis with microscopy

Comparison of results from microscopy and nested PCR is summarized in Table 3. Microscopic examination of blood films reported cases of *P. falciparum* (180 positive cases), cases of *P. vivax* (131 positive cases) in all the study sites. Three mixed infections of *P. falciparum* and *P. vivax* were detected in the samples from Omo Nada. In relative terms the highest numbers of *P. falciparum* and *P. vivax* patients were reported from Omo Nada site. Of all 180 microscopically confirmed *P. falciparum* cases, 111 cases (62%) were mono-infections for *P. falciparum*, 44 cases (24%) were *P. vivax* mono-infections, 18 cases (10%) were double infections with *P. falciparum* and *P. vivax,* two cases (1%) were double infections with *P. falciparum* and *P. malariae,* and five cases (3%) were confirmed negative for any *Plasmodium* species by the nested PCR. Of all 131 microscopically confirmed *P. vivax* cases, 110 cases (84%) were *P. vivax* mono-infections, 14 cases (11%) were mono-infections for *P. falciparum*, two cases (2%) were detected for *P. malariae* mono-infection, three cases (2%) were double infections with *P. falciparum* and *P. vivax* and two cases (2%) were confirmed negative for any *Plasmodium* species by the nested PCR. Molecular detection by nested PCR identified that many of the *P. vivax* cases (44/180) were incorrectly diagnosed as *P. falciparum* by microscopy. A total of seven negative cases are observed. These seven negative cases were reconfirmed for their status by amplification for *Pfmdr1* and *Pfcrt* alleles. *P. ovale* was not detected either by microscopy or by nested PCR. In total, 23 mixed infections were detected by nested PCR compared to microscopy-based detection.

**Table 3:** Comparison of microscopy and nested PCR in the diagnosis of malaria in southern Ethiopia

**Microscopy**	**Nested PCR**						
	***P. falciparum***	***P. vivax***	***P. malariae***	***P. falciparum + P. vivax***	***P. falciparum + P. malariae***	**Negative**	**Total**
*P. falciparum*	111	44	0	18	2	5	180
*P. vivax*	14	110	2	3	0	2	131
*P. falciparum + P. vivax*	0	0	0	3	0	0	3
*Total*	125	154	2	24	2	7	314

*Pf = P. falciparum, Pv = P. vivax, Pm = P. malariae, Po = P. ovale, Pf + Pv = P. falciparum* and *P. vivax, Pf + Pm = P. falciparum* and *P. malariae*.

### Gametocyte identification using microscopy

Of all the 314 study subjects, 32 (10.2%) carried gametocytes. Of which, 17(5.5%) were *P. vivax* and 15 (4.8%) were *P. falciparum* gametocyte stage. All the gametocyte stages were identified from 307 study subjects positive for *Plasmodium* parasites.

### Phylogenetic analysis

**Figure 1:**
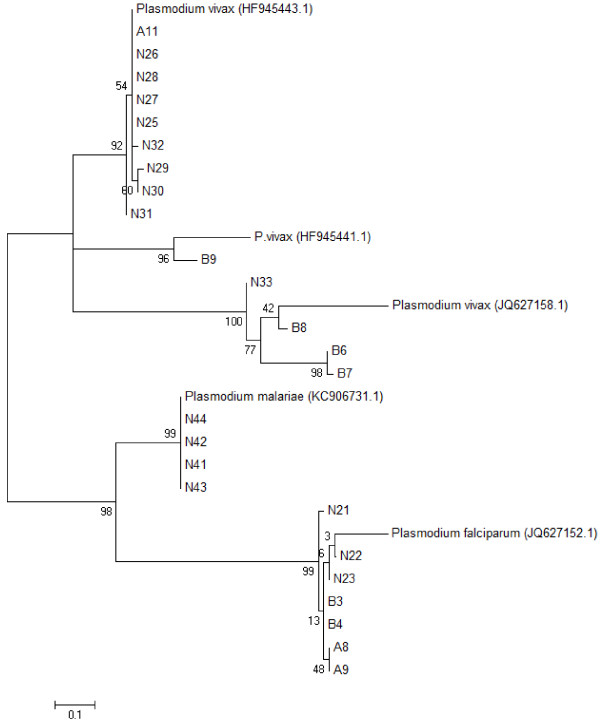
Reconstructed phylogenetic tree using 18S ribosomal RNA nucleotide sequence from Plasmodium field isolates.

The result of 31 Ethiopian specific isolates (11 *P. falciparum* isolates, 16 *P. vivax* isolates and four *P. malariae* isolates) originated from different geographical areas and sequenced for 18srRNA, is summarized in Table 4. The identified nucelotide substitutions including the insertions and deletions observed in different *Plasmodium* isolates is summarized in Table 5. The reconstructed phylogenetic tree inferred two distinct clades. All *P. vivax* isolates from Ethiopia matched in one group whereas all the *P. falciparum* and *P. malariae* clustered in another clade. Within the main clade, *P. vivax* clustered into three, *P. falciparum* clustered into two and *P. malariae* placed into one subcluster (Figure 1). The first subcluster with nine isolates contained 70 single nucleotide polymorphisms (SNP), the second subcluster included 150 SNPs and the third subcluster contained 53 SNPs.

**Table 4:** Parasite isolates utilized for phylogenetic analysis from the three study sites

	**Geographical regions**			
	**Omo Nada (ID)**	**Bala Wajo (ID)**	**Arba Minch (ID)**	**Total**
***P. falciparum***	3 (N21-N23)	2 (B3-B4)	6 (A3-A6, A8-A9)	11
***P. vivax***	9 (N25-N33)	4 (B6-B9)	3 (A10-A11, A17)	16
***P. malariae***	4 (N41-N44)	0	0	4

**Table 5:** **Ethiopian *Plasmodium *** **species with their single nucleotide polymorphism**

	***Plasmodium *** **no**	**Insertion**	**Deletion**	**Substitution**
	N21	1	3	18
Pf	A9	1	3	14
	N23	1	3	14
	B6	6	0	26
Pv sub cluster 1	B8	5	0	24
	B7	5	0	23
	N32	0	7	4
Pv sub cluster 2	N29	0	7	4
	A11	0	7	4
Pv sub cluster 3	B9	2	0	1

*Pf = P. falciparum, Pv = P. vivax, Pm = P. malariae, Po = P. ovale, Pf + Pv = P. falciparum* and *P. vivax, Pf + Pm = P. falciparum* and *P. malariae*.

## Discussion

Early diagnosis and prompt treatment is crucial in controlling malaria infections. Microscopic diagnosis and RDTs are widely used methods for malaria surveillance in Ethiopia. The most fatal species, *P. falciparum,* must be rightly differentiated from the other *Plasmodium* species (especially *P. vivax*), thereby directing febrile patients towards prompt treatment that can save lives. The rationale of the study was to assess species mismatch and the specificity of microscopy in differentiating *Plasmodium* spp. in regions where two virulent species of *Plasmodium,* namely *P. falciparum* and *P. vivax*, co-exist.

In Ethiopia, *P. falciparum* and *P. vivax* are the predominant parasite with a prevalence of 55 and 45%, respectively [23]. In most endemic regions across Ethiopia both *Plasmodium* co-exist. The present study report a prevalence of *P. vivax* (55%) remained high compared to *P. falciparum* (45%) during this season. The rationale of the study was to evaluate the limitations of microscopy in differentiating *Plasmodium* in Ethiopia that may contribute to improve individual’s life by an appropriate administration of anti-malarials. When slide positive cases were compared by molecular detection methodology, a high proportion of *P. vivax* individuals were misdiagnosed as *P. falciparum*-positive and were treated with AL, a drug of choice in treating *P. falciparum* infections. However the treatment of 14 cases (11%) microscopically diagnosed as *P. vivax* were mono-infections for *P. falciparum.* These results imply that these individuals were mistreated with chloroquine rather with AL. This may have had a considerable impact on treatment failure as they were misdiagnosed microscopically. Such a scenario may lead to severe malaria-related ailments and to the extent of mortality in these 14 cases. All the laboratories in the study area did not use buffer when preparing Giemsa staining solution. This might be one of the reasons for a high report of species mismatch and low sensitivity of microscopy. pH is an important component of Giemsa stain and pH 7.2 is recommended, as it highlights the Schüffner's dots of *P. vivax* and Maurer's clefts of *P. falciparum,* which are essential characteristics for malaria diagnosis by microscopy.

Additionally, microscopy methodologies failed to detect multiple infections. As demonstrated by a plethora of other studies that compared microscopy with molecular diagnosis [12,24-30], nucleic acid testing by the nested PCR approach provides an accurate differentiation of *Plasmodium*, and that helps to rule out false positives and mixed infections [10,31]. A high prevalence of mixed infections, especially with *P. falciparum* and *P. vivax,* irrespective of study sites were observed. Of the 21 individuals with double infections, three were reported as *P. vivax* and 18 were *P. falciparum*-positive by microscopy. The reason why microscopy failed to detect mixed infections could be due to the presence of higher numbers of parasites of one species relative to the other. Moreover, in laboratories with overwhelming numbers of patients, the workload is not proportional to the laboratory staff involved. Therefore, this leads the laboratory personnel not to utilize adequate time to examine the blood film.

Although *P. ovale* is one of the prevalent parasites in western Africa [32], no individuals infected with *P. ovale* were observed in any of the studied three sites. Additionally, *P. malariae,* a species which is less common in Ethiopia, was detected in four individuals with two as mono-infection and two as double infection with *P. falciparum*. A total of seven (2%) negative cases were observed. Of these individuals, five were treated for *P. falciparum* and two for *P. vivax*. The false positive cases were reconfirmed as negative for other *Plasmodium spp*. additionally; these false positive samples were subjected to subsequent amplification for both *pfmdr1* and *pfcrt* alleles. Microscopy failed to detect *P. malariae* mono-infections and mixed infections. The dominance of either *P. falciparum* or *P. vivax* can possibly be a reason for the failure to detect *P. malariae* by microscopy. In addition, the pattern of fever and duration of the erythrocyte 72-hour developmental cycle may contribute to *P. malariae* low numbers. Although microscopy remains as a gold standard, it has its limitations to differentiate *Plasmodium spp*., in addition to low parasite counts [33]. Factors influencing the precision of blood-smear diagnosis including the experience and motivation of technicians, smear quality, the inability to determine parasite species at low parasitemia, the loss of slide quality with time and the quality of the reagents. Artifacts resembling malaria are common. Currently RDTs are widely used in malaria surveillance programmes to differentiate only *P. falciparum* or *P. vivax* infections [34].

Many studies within Ethiopia reported conflicting results on the presence of *P. malariae* prevalence, including Federal Ministry of Health of Ethiopia. A few of these reported the presence only of *P. falciparum* and *P. vivax*[6]*,* whereas other reports demonstrated the prevalence of *P. malariae* in addition to the two dominant species in the country [35]. A study from Thailand reported that fever induced by low parasitaemia by *P. vivax* might limit parasitaemia and the pathogenic potential of *P. falciparum*[35]. In addition, the role of *P. malariae* in the aetiology of human glomerulonephritis is based on circumstantial clinical and epidemiological evidence and on renal biopsies showing granular immune deposits in the glomeruli [36].

Microscopic diagnosis of malaria is the most widely used approach for epidemiological studies. The blood smear provides much vital information that helps to correlate the parasite density to clinical malaria. Unfortunately, microscopy detection has both qualitative and quantitative limitations. Blood-smear microscopy reaches its limit of detection when parasitaemia falls below 40 infected red blood cells (IRBC) per μl of blood (10^8^ total body parasites), and the reproducibility of parasite counts and species identification is frequently inconsistent [37]. Factors influencing the precision of microscopy-based diagnosis largely depend on high experience, motivation of technicians, due diligence in examination over an adequate period of time, smear and slide quality, and differentiation from artifacts resembling malaria [38]. False positives are common in laboratories with high patient flow and when the numbers of laboratory personnel are not proportional to the laboratory workload. Difficulties are compounded when infections contain more than one parasite species. The other disadvantage of a false positive microscopic result is patients being exposed to drugs leading to unnecessary side effects and drug wastage. Patients will also be delayed from early treatment for other infections and that can be life threatening [39].

The phylogenetic relatedness of *P. vivax* and *P. falciparum* isolates is rather not restricted to one region, but seems to be widespread across different geographical regions within Ethiopia as the isolates from Abra Minch are also found in homology to isolates from Omo Nada. All isolates of *P. malariae* were represented from the site Omo Nada and were clustered together. Moreover, *P. vivax* showed a more diverse population than *P. falciparum* and *P. malariae*.

## Conclusions

False positivity, under-reporting of mixed infections and a significant number of species mismatch needs attention and should be improved for accurate diagnosis. Efforts must continue to re-train laboratory staff and provision of appropriate reagents based on the standard staining procedure for the diagnosis of *Plasmodium* parasite shall improve diagnosis. Overall, the finding of the current study highlights an important concern in the diagnosis of malaria by microscopy alone in southern Ethiopia and has important repercussions in understanding malaria epidemiology and subsequent control.

## Competing interests

The authors have declared that they have no competing interests.

## Authors’ contributions

SK designed and performed the field study and experiments with drafting first draft. NB, AA and GM contributed to the study design and study samples and revisions of MS. TPV designed the experiments, contributed to materials and tools, supervised the experiments, data analysis, writing of the MS. All authors read and approved the final manuscript.
